# Longitudinal Associations Between the Adolescent Family Environment and Young Adult Substance Use in Australia and the United States

**DOI:** 10.3389/fpsyt.2019.00821

**Published:** 2019-11-12

**Authors:** Jessica A. Heerde, Jennifer A. Bailey, John W. Toumbourou, Richard F. Catalano

**Affiliations:** ^1^Department of Paediatrics, The University of Melbourne, Parkville, VIC, Australia; ^2^Population Health Studies of Adolescents, Murdoch Children’s Research Institute, Parkville, VIC, Australia; ^3^Social Development Research Group, School of Social Work, University of Washington, Seattle, WA, United States; ^4^Centre for Social and Early Emotional Development, School of Psychology, Deakin University, Melbourne, VIC, Australia

**Keywords:** family environment, attachment, AUDIT, cannabis, adolescence, young adulthood, longitudinal study, cross-state study

## Abstract

**Introduction:** Harmful alcohol and cannabis use are social concerns associated with a range of negative outcomes. Prior research has identified links between disrupted parent-child attachment and child-adolescent substance use.

**Materials and Methods:** This study used cross-national data from the International Youth Development Study (IYDS; Victoria, Australia and Washington State, USA) to investigate the relationship between early adolescent family environment characteristics, mid-adolescent attachment to parents, and young adult harmful alcohol and cannabis use. The moderating role of state on these relationships was also tested. State-representative samples of students in Grade 7 (age 13, 2002) were recruited and followed longitudinally at ages 14, 15, and 25 (n = 1,945, 53% female, 50% in Victoria).

**Results:** Cross-state differences were evident in levels of family management, parent attitudes favorable to drug use, sibling alcohol and cannabis use, attachment to parents, and past year alcohol and cannabis use. Significantly higher rates of problematic alcohol use were reported by young adults in Victoria (25% vs. 14% in Washington State). Young adults in Washington State reported significantly higher rates of problematic cannabis use (14% vs. 10% in Victoria). Path modeling showed that characteristics of positive family environments (e.g., low conflict) in early adolescence were associated with higher attachment to parents and lower alcohol and cannabis use in mid-adolescence. Sibling substance use and more favorable parent attitudes to drug use were associated with past year alcohol and cannabis use in mid-adolescence. Results showed higher attachment to parents in mid-adolescence did not uniquely predict lower problematic alcohol or cannabis use in young adulthood. No significant cross-state differences in this pattern of associations were found.

**Discussion:** The implications of the current findings suggest that prevention and intervention strategies targeted at reducing problematic substance use into young adulthood may benefit from considering the influence of behavioral norms and attitudes in family relationships.

## Introduction

Preventing harms associated with substance use, including alcohol and cannabis, are international public health priorities. Population rates of heavy alcohol and cannabis use peak in early adulthood (1), meaning this period of development is a critical time for the emergence of substance use problems that represent preventable contributors to rates of morbidity and mortality among this age group (2, 3). To reduce the harmful effects of alcohol and other drug (substance) use, it is important to identify modifiable influences. One area of continued investigation is the link between disrupted parent-child attachment and later substance use (4–6). In this study, we analyze longitudinal data to identify modifiable influences that emerge from two theories of the development of substance use; *attachment* and *social development* theories.

Longitudinal studies offer the opportunity to understand those factors that influence problematic alcohol and cannabis use and provide a foundation from which to test the developmental effects of differing social contexts. Cross-national comparisons of longitudinal study findings offer additional benefits as they (1) permit testing of the role of macro-level policy and other contextual differences in alcohol and cannabis use and (2) promote understanding of the implications for feasible policy and prevention options. The observation of cross-national differences in as few as two countries, when predicted on the basis of theory, can result in highly interpretable empirical findings (7, 8).

The International Youth Development Study (IYDS) is a longitudinal research project that has conducted cross-state comparisons in the prevalence of alcohol and cannabis use, and its predictors, using data collected from state-representative samples of adolescents and young adults in Victoria, Australia and Washington State, United States (USA). At the study outset, Washington State and Victorian samples were similar in demographic and economic characteristics including population size, urbanization, educational participation, and prosperity (9). Standardized methodologies (sampling, recruitment, survey consent, and administration) were used in both states. Further, standardized measures of alcohol and cannabis use and other study variables were used in both states, and these measures were pilot tested to ensure comparability (9).

Thus, differences observed in alcohol and cannabis use or its predictors in the IYDS are likely to reflect real differences in policy and social contexts between the two states. Australia and the USA adopt different policy approaches aimed to reduce substance use among adolescents and young adults. Broadly, Australian policy focuses on minimizing the harms associated with young people’s substance use, whereas policies in the US encourage young people to abstain from substance use and apply punitive consequences as a deterrent to substance use through a zero-tolerance approach. Previous studies conducted using the IYDS data have shown adolescents and young adults in Victoria report higher rates of alcohol use (10, 9, 11) but lower rates of cannabis use compared to adolescents and young adults in Washington State (10). Further, analyses using IYDS data provide evidence that cross-national differences exist in predictors for health and social problems such as substance use between Victorian and Washington State participants (10, 11), however the relationships between these predictors and problems are cross-nationally similar in multivariate analyses.

One approach to addressing the incidence of substance use and its adverse consequences on adolescent and young adult health and well-being is to understand developmental influences. Longitudinal studies can be analyzed to identify risk factors (that increase the probability of substance use) and protective factors (that decrease the probability of substance use or mediate or moderate the effect of risk factors; 12, 13). The family environment is cited as an important sphere of influence for preventing substance use (14). As such, developmental researchers have investigated risk and protective factors in the adolescent family environment, including attachment influences on substance use. Family risk factors that predict adolescent substance use include: conflict with family members (15); poor management strategies; substance use among family members, and favorable parent attitudes to substance use (15, 10, 16). Conversely, family protective factors against substance use include: attachment to parents and opportunities for prosocial behavior within the family environment (15, 17, 10).

The hypotheses to be tested in the current study are grounded in two conceptual perspectives: *attachment theory* (18, 19) and *social development* (the *Social Development Model; SDM*) (12). *Attachment theories* identify early problems in parent-child attachment as antecedents for later problems in social and emotional adjustment (18). The effects of attachment problems continue to be measured in later life (19). According to attachment theories, substance misuse arises in part due to social and emotional difficulties that originate from parent-child attachment problems.

The *SDM* is a theory of the socialization processes and the development of prosocial and antisocial behavior (12), including substance use. The SDM is distinct from attachment theories in explaining attachment to role models as the key factor in the development of adolescent substance use. It hypothesizes that individuals learn patterns of behavior (prosocial or antisocial), in multiple socializing contexts (family, peer-group, school, community). The SDM posits that individuals are socialized through perceived opportunities for involvement in activities and interactions with others, actual involvement and interaction, skills to participate in these involvements and interactions, and rewards or costs perceived from these involvements and interactions. Involvement that is rewarded encourages development of a social bond between individuals and the socializing context; this bond influences behaviors because individuals are motivated to conform to the norms and values of the socializing unit.

The current paper, informed by both *attachment theory* (18, 19) and the *Social Development Model* (12) seeks to investigate the relationship between early adolescent family environment characteristics, mid-adolescent attachment to parents and substance use, and problematic alcohol and cannabis use in young adulthood. On the basis of these two theories, we hypothesize that (1) mid-adolescent attachment to parents will decrease problematic alcohol and cannabis use young adulthood; and (2) characteristics within individual and family contexts in adolescence will influence young adult problematic alcohol and cannabis use. The moderating role of state in associations between attachment and problematic alcohol and cannabis use will also be explored.

## Methods

### Participants

Data were drawn from young adults participating in an ongoing longitudinal study, the IYDS. The IYDS explores the development of healthy and problematic behaviors among adolescents and young adults from Victoria, Australia and Washington State in the United States (USA). The study began in 2002, and used a two-stage cluster sampling approach: public and private schools with Grades 5, 7, and 9 were randomly selected for recruitment into the study using a probability proportionate to grade-level size sampling procedure (20); and (2) one class at the appropriate grade level was randomly selected within each school (9) yielding samples of approximately 1,000 students at each grade level in each state. The original sampling and recruitment methods for the IYDS have been previously described in detail (9). In summary, across Grades 5, 7, and 9, 3,856 eligible students in Washington State and 3,926 students in Victoria were approached. Of these 2,885 participants (74.8%) in Washington State and 2,884 (73.5%) in Victoria consented to and participated in the 2002 survey. Participants have been followed longitudinally from 2002, with assessments at ages 12 through 18 years, 20 years, 22 years, and 25 years (in 2014). Retention rates across the study have remained high, with 98% retention in 2003 and 2004, 85% in 2008, 84% in 2010–11, 83% in 2012–13, and 87% in 2014–15 (21).

The current study analyzes data collected from participants in the 7th grade cohort, extracted from early-mid adolescence (Grade 7, Grade 8, Grade 9) and young adulthood (Age 25 years). The 7th grade cohort was the cohort chosen for long-term follow-up in the USA, and therefore has the most complete data in both Victoria and Washington State at each of the included timepoints. The analysis sample includes 1,945 participants (n = 984 in Victoria). At Grade 7, 51% of the sample were female and ranged in age between 12 and 16 years (mean (M) = 13 years, standard deviation (SD) = .43). At the age 25 time point, the sample ranged in age between 23 and 27 years (M[SD] = 25.14[.84]) and female participants formed 53% of the sample.

### Procedure

#### Survey Administration

The study design and measures (refer to *Instruments* section) were subjected to several processes in 2001 to ensure cross-national validity, including cognitive pretesting of the survey; pilot testing of the survey; and matching of sampling, recruitment, and survey administration procedures (9). Standardization ensured that method differences were minimized, overcoming problems with many international comparisons (22, 8). Trained survey staff used a single survey administration protocol in both states. At the study outset, written parental consent and participant assent was obtained for all participants. During formal schooling, surveys were administered to class groupings within schools. Following the completion of formal schooling, participants provided informed consent and the survey was completed online. The self-report survey took 50–60 min to complete. During adolescence, Victorian participants received a small gift (e.g., stress ball) and Washington State participants received USD$10, at the completion of each survey. Most recently, young adult participants in both states received a USD/AUD$40 gift voucher as reimbursement for their time.

### Instruments

The IYDS survey was adapted from the Communities That Care Youth Survey (23, 24). In 2001, in accordance with recommendations for cross-national instrument development (8), all survey measures underwent cognitive pretesting (9). This pretesting has been previously described in detail; in sum, this process included language review and cross-national item adaptation (9). The survey measures have demonstrated longitudinal validity and reliability when administered to participants in Victoria and Washington State (10, 11). Descriptive statistics for Grade 7 demographic characteristics, Grade7–Grade 8 family environment characteristics, Grade 9 measures of attachment to parents, past year alcohol and cannabis use, and young adult problematic alcohol and cannabis use (Age 25), including Cronbach’s Alpha, are provided in **Table 1**.

**Table 1:** Descriptive statistics for the study variables.

	Combined sample (CS; N = 1,945)		Washington State sample (WASH; n = 961)		Victorian sample (VIC; n = 984)		*p* value	Difference t / χ2	Cronbach’s alpha (α)		
	*M*(SD)	95% CI	*M*(SD)	95% CI	*M*(SD)	95% CI			CS	WASH	VIC
**Age 25 Problematic substance use**											
AUDIT (%, referent: low risk) Risky use Harmful/high risk use	19.71 4.04	– –	14.21 3.37	– –	25.00*** 4.69***	– –	<.0001	33.72	n/a	n/a	n/a
Cannabis (%, referent: low risk)	12.14	–	14.20*	–	10.14	–	.011	6.52	n/a	n/a	n/a
**Grade 7 Demographic characteristics**											
Family socioeconomic status	1.92 (.49)	[1.91, 1.95]	1.94 (.49)	[1.91, 1.97]	1.92 (.49)	[1.89, 1.95]	.344	.947	n/a	n/a	n/a
Female (%, referent: male)	50.59	–	50.63	-	50.56	–	.975	.001	n/a	n/a	n/a
Age (years)	13.01 (.41)	[12.99, 13.02]	13.09*** (.44)	[12.06, 12.12]	12.93 (.41)	[12.90, 12.95]	<.0001	8.37	n/a	n/a	n/a
Accommodation transitions (%, referent: no transitions)	25.99	–	27.08	–	24.92	–	.279	1.17	n/a	n/a	n/a
**Grade 7–8 Family environment characteristics**											
Low family conflict	2.79 (.70)	[2.76, 2.82]	2.77 (.70)	[2.73, 2.82]	2.81 (.70)	[2.77, 2.85]	.186	–1.324	.68	.66	.70
Family management	3.34 (.47)	[3.31, 3.36]	3.40*** (.47)	[3.37, 3.43]	3.28 (.46)	[3.25, 3.31]	<.0001	5.44	.70	.72	.69
Opportunities for prosocial involvement	3.12 (.64)	[3.09, 3.14]	3.11 (.67)	[3.06, 3.15]	3.12 (.62)	[3.09, 3.16]	.542	–.610	.72	.74	.6 9
Parental attitudes favorable toward drug use	1.35 (.45)	[1.33, 1.37]	1.22 (.38)	[1.20, 1.24]	1.47*** (.47)	[1.44, 1.50]	<.0001	–12.67	.57	.50	.57
Sibling alcohol use (%)	57.74	–	48.94	–	66.20***	–	<.0001	55.81	n/a	n/a	n/a
Sibling cannabis use (%)	19.96	–	25.08***	–	15.02	–	<.0001	28.97	n/a	n/a	n/a
**Grade 9 attachment to parents and substance use**											
Attachment to parents	2.84 (.72)	[2.81, 2.87]	2.82 (.73)	[2.77, 2.87]	2.85 (.71)	[2.81, 2.90]	.350	–.0935	.75	.74	.76
Past year alcohol use (%)	58.23	–	45.16	–	71.35***	–	<.0001	133.72	n/a	n/a	n/a
Past year cannabis use (%)	16.73	–	21.68***	–	11.75	–	<.0001	33.59	n/a	n/a	n/a

α, Cronbach’s alpha. n/a, scales with one item and therefore a Cronbach’s alpha could not be calculated. %, percent. χ^2^, chi-square. t, t-statistic. M, mean. SD, standard deviation. Female (coded 0 = male, 1 = female); Victoria (coded 0 = Washington State, 1 = Victoria); Accommodation transitions (coded 0 = no transitions, 1 = transitions); Sibling alcohol use (coded 0 = no use, 1 = recent use); Past year alcohol use (coded 0 = no use, 1 = recent use). Statistically significant state differences for continuous variables calculated using independent t-tests. Statistically significant state differences for dichotomous variables calculated using chi-square tests.

#### Demographic Characteristics

Demographic characteristics were measured in Grade 7. Participants reported their *age*, *gender*, and the *state* in which they lived (Victoria or Washington State). A measure of *family socio-economic status* was created using parent (mother and father) reported highest level of education (e.g., less than secondary school, completed secondary school, completed post-secondary school) and level of family income (ranging from less than $10,000 to $200,000+ per year). *Accommodation transitions* were measured using the item “Have you changed homes in the past year?” Response options ranged from “yes” (1) through to “no” (4) and were recoded to reflect “have not changed homes” (reference group) versus “changed homes on one or more occasion” (1) in the past year.

#### Early Adolescent Family Environment Characteristics

Five scales measured characteristics of the family environment in Grades 7 and 8. *Family conflict* was measured using three items. “People in my family have serious arguments” is an example item. Nine items, including “My family has clear rules about alcohol and drug use” were used to assess *family management*. For both scales, response options ranged from “definitely no” (1) to “definitely yes” (4) and were reverse coded such that higher scores indicated lower conflict and better management. Three items examined *opportunities for prosocial behavior* within the family environment. “If I had a personal problem, I could ask my mom or dad for help” is an example. Response options ranged from “definitely no” (1) to “definitely yes” (4). The scale measuring *parental attitudes favorable toward drug use* included four items, such as “How wrong do your parents feel it would be for you to use cannabis (pot, weed, grass)?” Response options ranged from “very wrong” (1) to “not wrong at all” (4). The influence of *sibling alcohol and cannabis use* was assessed using two items: “Have any of your brothers or sisters ever drunk alcohol (like beer, wine or liquor/spirits)?” and “Have any of your brothers or sisters ever used marijuana (pot, weed, grass)?”. Response options were dichotomous, “no” (reference group) and “yes” (1). Participant responses for early adolescent family environment characteristics were averaged to obtain a single scale score across the two waves (Grades 7 and 8).

#### Mid-Adolescent Attachment to Parents and Past Year Alcohol and Cannabis Use

*Attachment to parents* comprised four items administered in Grade 9, including “Do you feel very close to your mother?” and “Do you share your thoughts and feelings with your father?”. Response options ranged from “definitely no” (1) to “definitely yes” (4). *Alcohol use* in the past year at Grade 9 was examined using the item “In the past year (12 months), on how many occasions (if any) have you had alcoholic beverages (like beer, wine or liquor/spirits) to drink—more than just a few sips?”. The item “In the past year (12 months), on how many occasions (if any) have you used cannabis (pot, weed, grass)?” was used to measure *cannabis use* in the past year. Both items were rated on eight-point scales ranging from “never” (1) through to “40+ times” (8) and recoded to reflect “not at all” (reference group) versus “one or more occasions” (1) in the past year.

#### Young Adulthood Problematic Alcohol and Cannabis Use

*Problematic alcohol use* was measured at age 25 using the 10-items comprising the Alcohol Use Disorders Identification Test (AUDIT; 25). “How often during the last year have you found that you are unable to stop drinking once you had started?” and “How often during the last year has a relative, a friend, or a doctor or other health worker been concerned about your drinking or suggested that you cut down on your drinking?” are example items. Items were answered on a five-point scale of “never” (1), “monthly or less” (2), “2–4 times a month” (3), “2–3 times a week” (4), or “4 or more times a week” (5). Participants reporting no lifetime and no past year alcohol use were included as “never” for problematic alcohol use. Scores across all scale items were summed to form a total AUDIT score (0–35), where higher scores indicated more problematic alcohol use. Total scores were then recoded as per established guidelines into “low risk,” “risky,” “harmful,” and “high risk” alcohol use. Given the low prevalence of high-risk use in the current sample, harmful, and high-risk categories were combined. This is not uncommon with non-clinical samples. The final recoded AUDIT variable reflected levels of problematic use as being “low risk” (0), “risky use” (1), and “harmful/high risk” (2).

Nine items were used to measure *problematic cannabis use*. “Over the past year (12 months) how often has your use of marijuana caused you to feel anxious or depressed?” and “Over the past year (12 months) how often has your use of marijuana caused you to feel you couldn’t get through the week without it?” are example items. Each item was rated on an eight-point scale ranging from “never” (1) through to “40+ times” (8). Participants reporting no lifetime or past year cannabis use were included as “never” for problematic cannabis use. Scores across all scale items were summed to form a total problematic cannabis use score (0–27), where higher scores indicated more problematic cannabis use. Total scores were then categorized as per established guidelines (26) into “low risk,” “risky,” “harmful,” and “high risk” cannabis use. Given the low prevalence of participants in high and harmful risk categories, the item was recoded to reflect “no risk” (reference group) versus “risky use” (1).

### Statistical Analysis

The initial set of analyses were performed using Stata IC software for Windows (27), version 15.1. Cross-national differences in means and frequencies for all measures were examined using *t*-tests and chi-square analyses. Pooled standard deviations (28) were used to calculate effect sizes. Correlation analyses were performed to show highly correlated pairs or sets of variables that might result in collinearity in the multivariate analyses.

A series of longitudinal path models were estimated using Mplus, version 8.2 (29). Models 1 and 2 tested the hypothesized relationship between early adolescent family environment characteristics (Grades 7–8), mid-adolescent attachment to parents and past year substance use (Grade 9), and young adult AUDIT score (Age 25; Model 1) and problematic cannabis use (Model 2) use. Correlations between exogenous early adolescent family environment characteristics were not estimated in the model, however the observed correlations between these variables are taken into account by Mplus. Full information maximum likelihood estimation was used in all analyses to minimize potential bias due to missing data (29, 30). Demographic factors were included in the analysis. Model fit indices were examined in accordance with current recommendations (31, 32). The analyses presented here are fully standardized.

The results of Models 1 and 2 in the combined Victorian-Washington State sample were compared using multiple-group modeling to test the equivalence of the models across both states. Chi-square difference testing examined moderation by state. Differences in the constrained and unconstrained models were tested using the *difftest* function.

## Results

### State Comparisons of the Study Variables

**Table 1** presents the state comparisons of means and frequencies for demographic variables, Grade 7–8 family environment characteristics, Grade 9 attachment to parents and substance use, and AUDIT scores and problematic cannabis use in young adulthood (Age 25). Across the demographic variables, adolescents in Washington State were slightly older than those in Victoria at Grade 7. State level differences were clear for several Grade 7–8 family environment characteristics. Results showed more positive family management practices and higher rates of sibling cannabis use among Washington State compared to Victorian participants. More favorable parent attitudes to drug use and higher rates of sibling alcohol use were found for participants in Victoria. Regarding Grade 9 attachment and substance use, Washington State compared to Victorian adolescents showed higher levels of attachment to parents and past year cannabis use. Rates of past year alcohol use were greater for Victoria compared to Washington State adolescents. Results showed that at Age 25, Victorian young adults reported higher AUDIT scores (problematic alcohol use) compared to Washington State young adults. Conversely, young adults in Washington State reported higher rates of problematic cannabis use compared to those in Victoria.

### Correlations Between the Study Variables

**Table 2** presents the correlation matrix for all study variables. Intercorrelations between all study variables were low-moderate and in the expected direction. More favorable family management practices in early adolescence (Grade 7–8) were correlated with lower AUDIT scores and problematic cannabis use. With the exception of the association between sibling alcohol and cannabis use, intercorrelations between the analyzed early and mid-adolescent variables did not show multicollinearity, with no correlations >.80. Young adult AUDIT scores were most strongly correlated with gender, living in Victoria and Grade 9 past year alcohol use. Problem cannabis use in young adulthood was most strongly correlated with gender. The correlation between young adult AUDIT scores and problematic cannabis use was low (*r* = .21). As sibling alcohol and cannabis use variables were used in separate path models, both variables were retained for analysis.

**Table 2:** Zero-order correlations among study variables.

	1	2	3	4	5	6	7	8	9	10	11	12	13	14	15	16
1. Age 25 AUDIT	–	**.21**	.02	**–.17**	–.01	.001	**.13**	–**.06**	–.13	–**.06**	.04	**.07**	**.11**	–.03	**.13**	**.10**
2. Age 25 Problem cannabis use		–	**–**.01	**–.22**	.02	.04	–**.13**	–**.11**	–**.17**	–**.13**	**.08**	**.12**	.34	–**.06**	**.13**	**.41**
3. G7 Family SES			–	**–**.02	.05	–.02	–.02	.01	–.01	.003	–.04	–.01	–.01	–.02	–.01	.04
4. G7 Female				–	–**.13**	–.05	–.001	–**.09**	**.10**	–.03	–.01	**.06**	.04	–**.14**	.05	–.05
5. G7 Age					–	–.01	–**.19**	–.001	–**.06**	–.03	.02	**.06**	**.08**	–.02	.003	.04
6. G7 Accommodation transitions						–	–.04	–.04	–.04	–.01	.03	–.01	.05	–.01	.04	**.13**
7. G7 Victoria							–	.03	–**.12**	.01	**.28**	**.27**	–**.22**	.02	**.41**	–**.25**
8. G7–G8 Low family conflict								–	**.31**	**.46**	–**.21**	–**.24**	–**.23**	**.32**	–**.17**	–**.17**
9. G7–G8 Family Management									–	**.59**	–**.49**	–**.24**	–**.25**	**.28**	–**.29**	–**.26**
10. G7–G8 Family opportunities for prosocial behavior										–	–**.22**	–**.20**	–**.20**	**.48**	–**.17**	**–.21**
11. G7–G8 Parental attitudes favorable to drug use											–	**.26**	**.18**	–**.15**	**.34**	**.21**
12. G7–G8 Sibling alcohol use												–	**.80**	–**.13**	**.48**	**.30**
13. G7–G8 Sibling cannabis use													–	–**.11**	**.37**	**.59**
14. G9 Attachment to parents														–	–**.15**	–**15**
15. G9 Past year alcohol use															–	**.63**
16. G9 Past year cannabis use																–

Statistically significant associations in bold (at least p < .05). G7 = Grade 7, G8 = Grade 8, G9 = Grade 9. Female (coded 0 = male, 1 = female); Victoria (coded 0 = Washington State, 1 = Victoria); Accommodation transitions (coded 0 = no transitions, 1 = transitions); Sibling alcohol use (coded 0 = no use, 1 = recent use); Past year alcohol use (coded 0 = no use, 1 = recent use). Point biserial correlations were performed between a dichotomous variable and a continuous variable. Tetrachoric correlations were performed between two dichotomous variables. Pearson correlations were performed between two continuous variables.

### Path Model Findings

Two path models were estimated to examine the hypothesized relationship between early adolescent family environment characteristics, mid-adolescent attachment to parents and past year substance use, and young adult AUDIT scores (Model 1, **Table 3**), and problematic cannabis use (Model 2, **Table 4**).

**Table 3:** Path models predicting mid-adolescent attachment to parents and past year alcohol use and young adult AUDIT scores.

Association estimated	Standardized estimate	Unstandardized estimate (SE)	*p*-value
G9 Attachment predicted by G7-G8:			
Low family conflict	.097***	.023	.032
Family management	– .032	.028	.264
Family opportunities for prosocial behavior	.440***	.025	<.0001
Parent attitudes favorable toward drug use		.024	.076
Sibling alcohol use (referent: no use)	– 0.15	.023	.513
Family socioeconomic status^	– .027	.022	.234
Female (referent: male)^	– .119***	.022	<.0001
Age (years)^	– .027	.021	.182
Accommodation transitions (referent: no transitions)^	– .006	.020	.779
Victoria (referent: Washington State)^	.017	023	.462
G9 Past year alcohol use predicted by G7-G8:			
Low family conflict	– .067*	.031	.031
Family management	– .176***	.039	<.0001
Family opportunities for prosocial behavior	– .008	.038	.822
Parent attitudes favorable toward drug use	.238***	.033	<.0001
Sibling alcohol use (referent: no use)	.205***	.027	<.0001
Family socioeconomic status^	– .006	.028	.845
Female (referent: male)^	.038	.028	.171
Age (years)^	.003	.026	.919
Accommodation transitions (referent: no transitions)^	.032	.027	.247
Victoria (referent: Washington State)^	.198***	.029	<.0001
Age 25 AUDIT predicted by G9:			
Attachment	– .028	.036	.436
Past year alcohol use	.177***	.051	<.0001
Family socioeconomic status^	.037	.033	.266
Female (referent: male)^	– .238***	.034	<.0001
Age (years)^	.014	.037	.697
Accommodation transitions (referent: no transitions)^	– .007	.034	.835
Victoria (referent: Washington State)^	.141***	.040	<.0001
Correlations specified in the model:			
G9 Attachment with G9 Past year alcohol use	– .104**	.032	.001

G7 = Grade 7, G8 = Grade 8, G9 = Grade 9. Correlations among exogenous G7-G8 early adolescent family environment characteristics and demographic variables are not estimated in the model; the observed correlations between these variables are taken into account by Mplus. SE = standard error. ^Demographic factors measured at G7. Female (coded 0 = male, 1 = female); Victoria (coded 0 = Washington State, 1 = Victoria); Accommodation transitions (coded 0 = no transitions, 1 = transitions); Sibling alcohol use (coded 0 = no use, 1 = recent use); Past year alcohol use (coded 0 = no use, 1 = recent use). Statistically significant results indicated with asterisks: *p < .05, **p < .01, ***p < .001.

**Table 4:** Path models predicting mid-adolescent attachment to parents and past year cannabis use and young adult problematic cannabis use.

Association estimated	Standardized estimate	Unstandardized estimate (SE)	*p*-value
G9 Attachment predicted by G7-G8:			
Low family conflict	.100***	0.23	<.0001
Family management	-.030	.029	.301
Family opportunities for prosocial behavior	.441***	.025	<.0001
Parent attitudes favorable toward drug use	-.047	.024	.056
Sibling alcohol use (referent: no use)	.006	.023	.796
Family socioeconomic status^	-.026	.023	.255
Female (referent: male)^	-.120***	.022	<.0001
Age (years)^	-.029	.021	.164
Accommodation transitions (referent: no transitions)^	-.007	.020	.740
Victoria (referent: Washington State)^	015	.023	.512
G9 Past year alcohol use predicted by G7-G8:			
Low family conflict	-.047	.037	.203
Family management	-.165***	.045	<.0001
Family opportunities for prosocial behavior	-.061	.044	.160
Parent attitudes favorable toward drug use	.171***	.036	<.0001
Sibling alcohol use (referent: no use)	.299***	.029	<.0001
Family socioeconomic status^	.072*	.031	.019
Female (referent: male)^	-.048	.035	.169
Age (years)^	-.024	.036	.503
Accommodation transitions (referent: no transitions)^	.061	.033	.064
Victoria (referent: Washington State)^	-.220***	.036	<.0001
Age 25 AUDIT predicted by G9:			
Attachment	-.009	.045	.842
Past year alcohol use	.394***	.055	<.0001
Family socioeconomic status^	-.026	.042	.534
Female (referent: male)^	-.199***	.044	<.0001
Age (years)^	-.028	.049	.574
Accommodation transitions (referent: no transitions)^	-.028	.042	.496
Victoria (referent: Washington State)^	-.042	.050	.406
Correlations specified in the model:			
G9 Attachment with G9 Past year alcohol use	-.080*	.039	.041

G7 = Grade 7, G8 = Grade 8, G9 = Grade 9. Correlations among exogenous G7-G8 early adolescent family environment characteristics and demographic variables are not estimated in the model; the observed correlations between these variables are taken into account by Mplus. SE = standard error. ^Demographic factors measured at G7. Female (coded 0 = male, 1 = female); Victoria (coded 0 = Washington State, 1 = Victoria); Accommodation transitions (coded 0 = no transitions, 1 = transitions); Sibling cannabis use (coded 0 = no use, 1 = recent use); Past year cannabis use (coded 0 = no use, 1 = recent use).

#### Young Adult Audit Scores

The first model, testing the relationship between family environment characteristics, attachment to parents and AUDIT scores, showed good fit [χ^2^(5, *N* = 1,698) = 16.44, *p* = .0057, comparative fit index (CFI) = .978, Tucker-Lewis index (TLI) = .856, root-mean-square error of approximation (RMSEA estimate) = .037]. Lower levels of family conflict and greater opportunities for prosocial behavior within the family environment in early adolescence (Grade 7–8) significantly predicted greater attachment to parents in Grade 9. Being female was uniquely associated with lower Grade 9 attachment to parents. Lower past year alcohol use at Grade 9 was predicted by less family conflict and more positive family management practices in early adolescence (Grade 7–8). Both sibling alcohol use and adolescents’ perceptions of parents more favorable attitudes toward drug use, showed significant associations with past year alcohol use. Living in Victoria emerged as a unique predictor of Grade 9 past year alcohol use. Living in Victoria and higher Grade 9 past year alcohol uniquely predicted age 25 AUDIT scores. Being female predicted lower AUDIT scores in young adulthood. Adolescent family attachment was not significantly related to age 25 AUDIT scores.

#### Young Adult Problematic Cannabis Use

**Table 4** shows results from the model testing relationships between family environment characteristics, attachment to parents and problematic cannabis use. The data fit the model well [χ^2^(5, *N* = 1,698) = 5.270, *p* = .3838, CFI = .999, TLI = .996, RMSEA = .006]. Lower levels of family conflict and greater opportunities for prosocial behavior in the family environment in early adolescence predicted attachment to parents in Grade 9. Attachment was negatively related to female gender. More past year cannabis use at Grade 9 was predicted by parent attitudes favorable toward drug use, sibling cannabis use, and lower family socioeconomic status, whereas living in Victoria and early adolescent positive family management practices predicted lower past year cannabis use in Grade 9. Grade 9 past year cannabis use uniquely predicted age 25 problematic cannabis use. Being female predicted lower problematic cannabis use in young adulthood. Adolescent attachment to parents was not related to later cannabis problems.

### Tests of Cross-State Equivalence

Multiple-group modeling revealed no significant cross-country differences in the pattern of associations specified in Model 1 or Model 2.

## Discussion

Harmful alcohol and cannabis use are social concerns associated with a range of negative outcomes. The current longitudinal study, using data from the International Youth Development Study, has tested attachment theory and the SDM to investigate the relationship between early adolescent family environment characteristics, mid-adolescent attachment to parents and substance use, and problematic alcohol and cannabis use in young adulthood. We found cross-state differences in levels of problem alcohol and cannabis use in young adulthood. The rate of problem alcohol use (AUDIT scores) among young adults in Victoria was higher than in Washington State. Conversely, rates of problem cannabis use among young adults in Washington State were greater than in Victoria. Some cross-state differences in levels of early adolescent family characteristics and mid-adolescent attachment to parents were found. Consistent with prior literature suggesting developmental differences in trajectories of substance use where males compared to females show higher rates of substance use into early adulthood (33, 34, 11), we found being female predicted lower AUDIT scores and problem cannabis use in young adulthood. Despite the observed level differences across countries, the current results showed no statistically significant cross-state difference in longitudinal associations between family environment measures and either problematic alcohol or cannabis use. These findings suggest that family risk and protective factors may exert a cross-nationally similar effect on the development of young adult substance use. Further cross-national research examining the longitudinal effects of family environment characteristics should seek to confirm the current findings and investigate characteristics in other potential spheres of influence (e.g., peer-group, community).

Our findings supported the hypotheses that characteristics of the family environment and adolescent substance use would be associated with problematic alcohol and cannabis use in young adulthood. The findings of this study are similar to those reported in previous studies, such that less positive family environment characteristics (e.g., family conflict) were associated with later substance use (e.g., 15, 10, 16). Importantly, the current findings extend over a longitudinal period of over 12 years and thus are intrinsically valuable in contributing to understanding of the long-term developmental influence of the family environment on trajectories of substance use. The current findings suggest a developmental process in both states whereby early adolescent family factors predict Grade 9 alcohol and cannabis use, which is then maintained into young adulthood.

Although prior longitudinal studies have reported higher levels of attachment to parents are associated with lower rates of substance use, our results did not support the hypotheses that adolescent attachment to parents would be associated with less problematic alcohol and cannabis use in young adulthood. Attachment theory has long suggested that early problems in parent-child attachment are antecedents for later social and emotional adjustment problems (18), including substance use. Measures of early childhood family attachment were not available in the current study; hence, we were unable to test the potential prospective association between early life family-based attachment and young adult substance use. However, in line with SDM theory (12) and suggestions that the family environment is pivotal in substance use prevention (14), we found that early adolescent family conflict, parental norms, and sibling substance use were key predictors of later adolescent substance use and, by extension, problem use of alcohol and cannabis in young adulthood in both Victoria and Washington. We also found a small effect of family socioeconomic status on young adult cannabis use. Similar findings have been reported elsewhere (35– 37). Further investigations on the effect of early economic deprivation and poverty, and broader environmental influences, on later substance use are warranted. In this context, our study findings are important for guiding the development of interventions targeting the adolescent family milieu and social norms within broader social contexts (e.g., peer-group, community).

Results supported the Social Development Model (SDM). Parent’s attitudes to substance use and the substance use behavior of siblings were found to predict adolescent and young adult alcohol and cannabis use. These findings align with the SDM proposition that the behavioral norms and attitudes of people that children and young people form social attachments to are the critical drivers in the development of health and social behavior (12). Our findings also suggest that higher rates of alcohol and cannabis use identified in the IYDS cohorts during adolescence (10, 38) are continued into early adulthood (11).

The current findings suggest that prevention and intervention strategies targeted at reducing substance use into young adulthood, including problematic alcohol and cannabis use, need to consider the influence of behavioral norms and attitudes in social relationships between family members from early on in adolescence. The lack of cross-state differences also suggests that common interventions targeting similar family environment characteristics (risk and protective factors) might be selected to reduce young adult substance use (alcohol and cannabis) in both states. It is also critically important to understand predictors and mechanisms of persistence and desistence of both alcohol and cannabis use across a range of spheres of influence (e.g., peer group, community) into and during adulthood.

### Strengths and Limitations of This Study

#### Study Strengths

Several strengths to the current study are noted. At the time of study commencement in 2002, the recruited sample was state representative, demonstrated high responses rates, and comprised approximately equal numbers of male and female participants. The study is unique in analyzing two cross-state samples, recruited, surveyed, and longitudinally followed using identical methods with high response rates (9). To young adulthood, the study has achieved strong participant retention. This study has detailed data on a wide range of risk and protective factors from early in adolescence and into young adulthood known to influence the development of healthy and problematic behaviors in adolescents, including those related to the family environment and participants’ use of substances. Therefore, the current study presents a unique opportunity to examine predictors of attachment and prospective associations between attachment and substance use, over multiple periods of development relative to prior studies. Thus, a noteworthy strength of this study is its ability to maximize the available data to investigate the current research questions and contribute vital knowledge to theories of development and attachment.

#### Study Limitations

Despite these notable strengths, several limitations to the study are acknowledged. The study results are generalizable only to states with similar school contexts and grade levels to those examined here. Measures of family environment characteristics, attachment, and substance use were based on self-report data. The use of self-report data in studies of adolescents and for the measures examined in this study is considered reliable (39). The factor structure of these measures has been validated (24) and these measures have shown adequate reliability and longitudinal validity in Victorian (10, 11) and Washington State (24) samples.

## Conclusions

Problematic alcohol and cannabis use are associated with negative health and social outcomes. Our study, using data from the International Youth Development Study, sought to identify modifiable influences that emerge from two theories of the development of substance use; *attachment* and *social development* theories. Our findings suggested that characteristics of the family environment, including family behavioral norms and attitudes, are important influences on substance use in adolescence and into young adulthood. These influences, as well as broader influences within social settings in which adolescents and young adults interact, are important in the development of substance use prevention and intervention strategies.

## Data Availability Statement

Please contact study directors regarding data availability. Requests to access the datasets should be directed to john.toumbourou@deakin.edu.au.

## Ethics Statement

The University of Melbourne Human Ethics in Research Committee and the Royal Children’s Hospital Ethics in Human Research Committee provided approval for this study in Australia. The University of Washington Human Subjects Institutional Review Board provided approval for the study in the USA.

## Author Contributions

JH, JB, and JT contributed to the conception and design of the study. JH performed the statistical analysis and wrote the first draft of the manuscript. JB assisted with the statistical analysis. JB and JT wrote sections of the manuscript. All authors (JH, JB, JT, RC) contributed to manuscript revision, read and approved the submitted version.

## Funding

JH is supported by a Research Fellowship provided through the Westpac Scholars Trust. The authors are grateful for the financial support of the National Institute on Drug Abuse (R01DA012140), the National Institute on Alcoholism and Alcohol Abuse (R01AA017188), the National Health and Medical Research Council (NHMRC; 491241) and the Australian Research Council (DP109574, DPO663371 and DPO877359) for supporting the IYDS. The content is solely the responsibility of the authors and does not necessarily represent the official views of the funders. The funding agencies did not have any involvement in the analysis and interpretation of data, the writing of the article or the submission of the article for publication.

## Conflict of Interest

JT is a director of the not-for-profit company Communities That Care Ltd that distributes the Communities That Care Youth Survey in Australia.

The remaining authors declare that the research was conducted in the absence of any commercial or financial relationships that could be construed as a potential conflict of interest.
